# oxLDL-induced aortic valve inflammation and calcification: opportunities for clinical translation

**DOI:** 10.1093/cvr/cvaf166

**Published:** 2025-09-23

**Authors:** Yu-Jen Wang, Michael A Matter, Christian M Matter

**Affiliations:** Center for Translational and Experimental Cardiology (CTEC), Department of Cardiology, University Hospital Zurich and University of Zurich, University of Zurich, Zurich, Switzerland; Center for Translational and Experimental Cardiology (CTEC), Department of Cardiology, University Hospital Zurich and University of Zurich, University of Zurich, Zurich, Switzerland; Department of Cardiology, University Heart Center Zurich, University Hospital Zurich, Raemistrasse 100, Zurich 8091, Switzerland; Center for Translational and Experimental Cardiology (CTEC), Department of Cardiology, University Hospital Zurich and University of Zurich, University of Zurich, Zurich, Switzerland; Department of Cardiology, University Heart Center Zurich, University Hospital Zurich, Raemistrasse 100, Zurich 8091, Switzerland

**This editorial refers to ‘OxLDL-induced FOXS1 mediates cholesterol transport dysfunction and inflammasome activation to drive aortic valve calcification’, by C. Jiang *et al.*, https://doi.org/10.1093/cvr/cvaf159.**

## 1. Calcific aortic valve disease—lack of drug treatment

Calcific aortic valve disease (CAVD) represents one of the most prevalent cardiovascular diseases in the aging population.^1^ It can progress to relevant aortic stenosis and accounts for significant cardiac morbidity and mortality. Current European guidelines on the treatment of aortic valve stenosis^2^ focus on surgical or interventional treatment modalities that comprise valvular repair or replacement. Although very effective, those modalities confer obvious risk and morbidity given their invasiveness. Additionally, they are directed against clinically, functionally and/or morphologically advanced CAVD.

**Figure 1:**
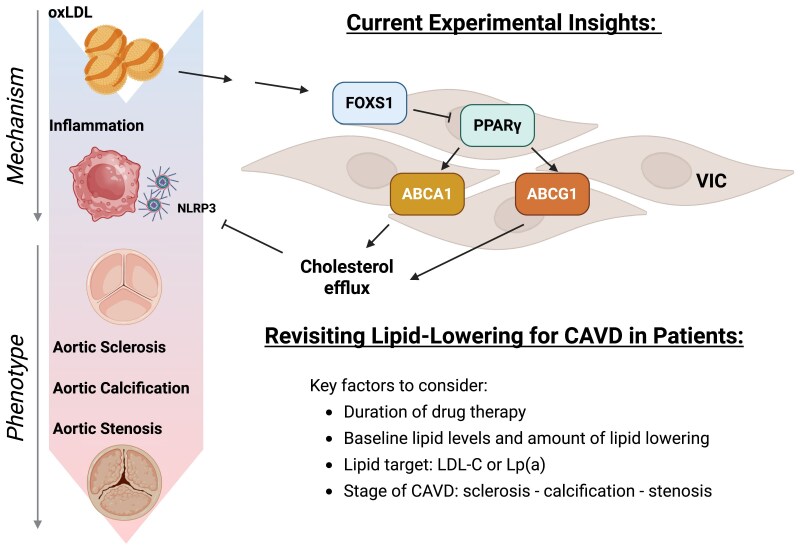
Dyslipidaemia and inflammation are critical contributors to experimental aortic valve calcification and subsequent stenosis. Currently, no pharmacological therapy is available to delay the progression of aortic valve disease in patients. Previous lipid-lowering trials failed to demonstrate clinical benefit. Jiang *et al*. now report that oxLDL induces FOXS1 expression in VIC, which suppresses PPARγ activity, impairs cholesterol efflux (via ABCA1/ABCG1), and activates the NLRP3 inflammasome. This novel mechanistic insight prompts a re-evaluation of lipid-lowering strategies in patients with aortic sclerosis up to calcific aortic valve stenosis. In particular, factors such as duration of drug therapy, baseline lipid levels and dosing regimens of lipid-lowering therapies, the targeted lipid species (e.g. LDL-C and Lp(a)), and aortic valve disease stage likely influence the therapeutic effects. oxLDL, oxidized low-density lipoprotein; FOXS1, forkhead box S1; PPARγ, peroxisome proliferator-activated receptor γ; ABCA1, ATP binding cassette subfamily A member 1; ABCG1, ATP-binding cassette subfamily G member 1; NLRP3, nucleotide-binding domain, leucine-rich-containing family, pyrin domain-containing-3; VIC, valvular interstitial cell; CAVD, calcific aortic valve disease; LDL-C, low-density lipoprotein cholesterol; Lp(a), lipoprotein(a).

To date, there is no approved drug treatment for CAVD. The recommendations in the current guidelines are limited to treatment of cardiovascular comorbidities.^3^ Notably, the role of lipid-lowering agents, in particular statins, is explicitly negated based on the negative results from two Phase 3 trials testing the effects of high-intensity statin on the progression of aortic valve stenosis.^4,5^ This leaves the question open, whether dysfunctional lipid metabolism is relevant in the early phases of aortic valve stenosis. The current study by Jiang et al.^6^ offers interesting new mechanistic insights, rekindling the spark of interest in potential drug treatment for CAVD (*Figure 1*).

## 2. oxLDL and inflammation in experimental calcific aortic valve disease

In this issue of Cardiovascular Research, Jiang et al. elucidated the molecular mechanisms by which oxidized low-density lipoprotein (oxLDL) exacerbated aortic valve calcification by activating the transcription factor Forkhead Box S1 (FOXS1). Initially, the authors identified increased expression of FOXS1 in valvular interstitial cells (VICs) of human calcified, stenotic aortic valves compared to normal ones. Ablation of FOXS1 mitigated valve calcification in both *ex vivo* human VICs and *in vivo* mouse models. Interestingly, transcriptomic data revealed a correlation between FOXS1 expression and cholesterol transport pathways. Applying knockdown and pharmacological strategies, they demonstrated that FOXS1 regulated the expression of the lipid transporters ATP binding cassette subfamily A member 1 (ABCA1) and ATP-binding cassette subfamily G member 1 (ABCG1), both established downstream targets of peroxisome proliferator activated receptor γ (PPARγ) in VICs. Decreased levels of these cholesterol transporters induced cholesterol accumulation and inflammatory responses (interleukin [IL]-1β, IL6, and NLRP3 [nucleotide-binding domain, leucine-rich-containing family, pyrin domaincontaining-3] inflammasome activation) associated with CAVD. Finally, they identified an upstream regulator BSCL2 (lipid droplet biogenesis associated, seipin) of PPARγ, highlighting its potential to treat CAVD by promoting cholesterol efflux.

Both dysregulation of lipid metabolism and inflammation play a role in atherosclerotic cardiovascular disease (ASCVD).^7^ However, the mechanisms underlying CAVD related to lipid dysregulation and inflammation are much less understood.3 Although differences are present, similar key concepts underlying the pathophysiology of atherosclerosis and CAVD may be presumed. Along this line, *in vitro* oxLDL stimulation and *in vivo* atherosclerosis-prone mouse models serve as important experimental tools for investigating molecular mechanisms of CAVD. Moreover, a proteomic-based comparison between human calcified atherosclerotic plaques and human calcified aortic valves revealed shared lipoprotein-related pathways between the two diseases, underscoring an association of lipid dysregulation with CAVD.^8^

## 3. Translating experimental insights of CAVD to the clinical context

Far less is known about the value of targeting lipid dysregulation and/or inflammation in CAVD than in ASCVD. An indicator towards a potential causal role of dyslipidaemia in progression of CAVD is presented by a study of subjects with heterozygous familial hypercholesterolemia, for whom an association between increased LDL–cholesterol and CAVD has been reported.^9^ Furthermore, genetic variation in the lipoprotein(a) [Lp(a)] locus, mediated by Lp(a) levels, has been identified as a causal factor for patients with aortic valve calcification.^10^ Lp(a) induced increased calcium deposition and the expression of pro-inflammatory cytokines.^11^ These findings suggest a causal role of LDL–cholesterol and Lp(a) for CAVD.

The present study offers interesting food for thought towards clinical translation (*Figure 1*). As mentioned above, several large-scale randomized controlled trials have shown no benefit of lipid-lowering treatment to prevent progression of aortic stenosis. However, those studies presented only relatively short-term median follow-up periods of 25 or 52.2 months, respectively.^4,5^ In terms of echocardiographic measures, CAVD typically progresses at an average rate of an increase in aortic velocity of about 0.16 m/s per year, and a decrease in valve area of 0.08 cm² per year.^12^ Once reaching a transvalvular aortic velocity of 2 m/s or more, progression to severe aortic stenosis typically occurs within 10 years. Thus, the time required to study a potential treatment effect of lipid-lowering therapies to prevent or decelerate progression of CAVD may be far in excess of what previous studies have investigated. Moreover, the magnitude of effects of lifelong genetic lipid changes on CAVD have to be placed in perspective to the duration of drug-based lipid-lowering.

Additionally, previous clinical studies in this field described a setting where at least mild CAVD is already present, i.e. a setting of secondary prevention. The current study by Jiang et al. demonstrates a role of lipid dysregulation and subsequent inflammation at the initiation and very early stages of CAVD. Translated into clinical application, the use of lipid-lowering or anti-inflammatory agents to prevent the onset or progression of CAVD in a primary prevention setting remains to be explored for LDL-C and inflammation (*Figure 1*)—or is being investigated for Lp (a) (NCT 05646381).

Extending the notions thus put forward beyond traditional markers of lipid dysregulation, there has been a recent surge in interest in the causal role of Lp(a) in both ASCVD and CAVD, as already mentioned above.^13^ Ongoing studies targeting Lp(a) promise important new insights and potentially new treatment options for both ASCVD and CAVD.

## 4. Conclusion and perspectives

New mechanistic insights into the pathophysiological processes underlying the initiation and progression of CAVD may unravel novel drug targets (*Figure 1*). The results put forward by Jiang et al. reignite the spark of recently dwindling interest in traditional lipid-lowering drugs to treat or prevent CAVD. Jiang et al. also provide fuel for thought on potential novel therapeutic targets, linking lipid dysregulation and inflammation, a concept which may prove fruitful within as well as beyond the setting of CAVD.
